# Heronapyrrole D: A case of co-inspiration of natural product biosynthesis, total synthesis and biodiscovery

**DOI:** 10.3762/bjoc.10.121

**Published:** 2014-05-26

**Authors:** Jens Schmidt, Zeinab Khalil, Robert J Capon, Christian B W Stark

**Affiliations:** 1 Fachbereich Chemie, Institut für Organische Chemie, Universität Hamburg, Martin-Luther-King-Platz 6, 20146 Hamburg, Germany; 2 Institute for Molecular Bioscience, The University of Queensland, St. Lucia, QLD 4072, Australia

**Keywords:** biomimetic synthesis, biosynthesis, heronapyrroles, microbial biodiscovery, natural products, nitropyrroloterpenes

## Abstract

The heronapyrroles A–C have first been isolated from a marine-derived *Streptomyces* sp. (CMB-0423) in 2010. Structurally, these natural products feature an unusual nitropyrrole system to which a partially oxidized farnesyl chain is attached. The varying degree of oxidation of the sesquiterpenyl subunit in heronapyrroles A–C provoked the hypothesis that there might exist other hitherto unidentified metabolites. On biosynthetic grounds a mono-tetrahydrofuran-diol named heronapyrrole D appeared a possible candidate. We here describe a short asymmetric synthesis of heronapyrrole D, its detection in cultivations of CMB-0423 and finally the evaluation of its antibacterial activity. We thus demonstrate that biosynthetic considerations and the joint effort of synthetic and natural product chemists can result in the identification of new members of a rare class of natural products.

## Introduction

**Figure 1:**
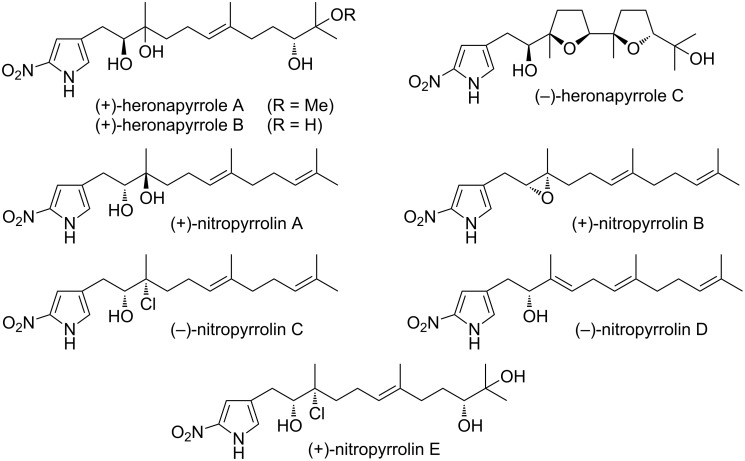
Known heronapyrroles A–C and nitropyrrolins A–E.

Heronapyrroles A–C (Figure 1) were first reported in 2010 by Capon et al. from a marine-derived *Streptomyces* sp. (CMB-M0423) obtained from a shallow water sand sample collected near Heron Island, Queensland, Australia (Figure 1) [1]. As first in class examples of natural products featuring a 2-nitropyrrole, further elaborated by a farnesyl side chain, the heronapyrroles exhibited promising antibacterial activity against *Staphylococcus aureus* (ATCC 2593 and 9144) and *Bacillus subtilis* (ATCC 6051 and 6633). Publication of the heronapyrroles was rapidly followed by an account of the biosynthetically related nitropyrrolins A–E, obtained from a different marine-derived actinomycete strain (CNQ-509) (Figure 1) [2]. Of note, the heronapyrroles and nitropyrrolins both feature the same unprecedented heterocyclic core (2-nitro-4-farnesylpyrrole) with closely related levels of oxidative functionalization of the farnesyl side chain.

**Scheme 1:**
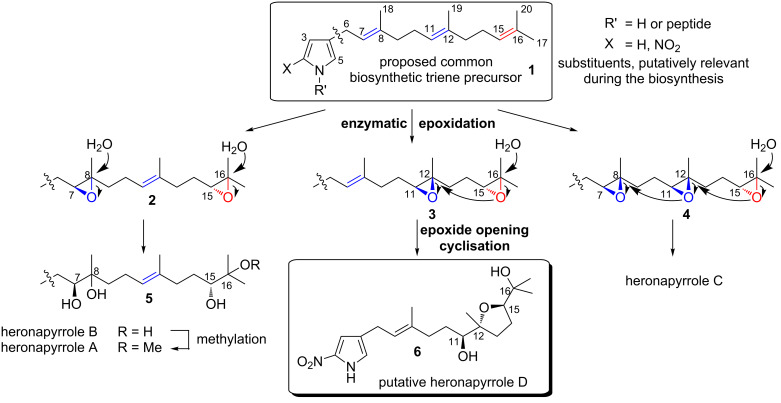
Plausible biosynthesis of heronapyrroles A–D.

In 2012 Stark et al. employed a biomimetic strategy to deliver the first asymmetric total synthesis of heronapyrrole C, confirming its constitution and establishing the relative as well as the absolute configuration [3–4] (recently, verified by Brimble et al. [5]). Building on these achievements, and in a departure from traditional natural products discovery, this report describes an innovative collaborative strategy that employed biosynthetic considerations, along with synthetic (Stark group) and natural products (Capon group) chemistry, to speculate on the likely existence of a hitherto unidentified heronapyrrole. This strategy culminated in the synthesis, detection and confirmation that heronapyrrole D (Scheme 1) was indeed a natural product.

## Results and Discussion

Although the genes responsible for heronapyrrole biosynthesis have not been identified yet, it is nevertheless possible to speculate on key aspects of this biosynthetic pathway, particularly those oxidative transformations that generate structural diversity [6–9] in the farnesyl side chain. For example, it is reasonable to conclude that 4-farnesylated 2-nitropyrrole **1** (Scheme 1) – arising from the action of a farnesyl transferase [10–11] on a suitable 2-nitropyrrole precursor – is a highly plausible common precursor for all heronapyrroles. Bisepoxidation of the Δ^7^ and Δ^15^ double bond in the farnesyl residue (for atom numbering see Scheme 1), followed by regio- and stereoselective nucleophilic addition of water (and methylation) would deliver heronapyrroles A and B. Similarly, regio- and stereoselective nucleophilic addition of water to a *tris*-epoxy farnesyl intermediate **4** could initiate a cyclization cascade [1,3,8] that would provide the *bis*-tetrahydrofuran heronapyrrole C (Scheme 1), via a mechanism that closely resembles polyether antibiotic biosynthesis [12–17]. This biosynthetic hypothesis raises the possibility that *Streptomyces* sp. (CMB-M0423) may produce an alternative *bis*-epoxy intermediate **3** that can deliver a hitherto undetected *mono*-tetrahydrofuran heronapyrrole (e.g., heronapyrrole D, Scheme 1). To test this hypothesis we completed an asymmetric synthesis of the putative natural product heronapyrrole D, and used this material to probe *Streptomyces* sp. (CMB-M0423) cultivations to test whether heronapyrrole D is indeed a natural product.

**Scheme 2:**
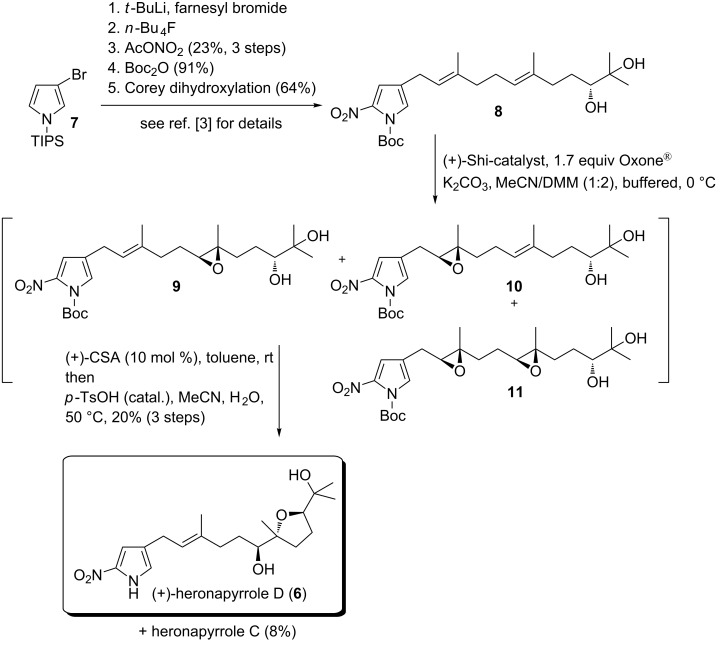
Synthesis of heronapyrrole D.

The asymmetric synthesis of heronapyrrole D commenced with enantiomerically pure diol **8** (Scheme 2) synthesized in five steps from commercially available TIPS-protected 3-bromopyrrole **7** [3]. This reaction sequence, which incorporates a regioselective pyrrole alkylation, an electrophilic aromatic nitration and a regio- and stereoselective Corey–Noe–Lin dihydroxylation [18], has previously been described (in the total synthesis of heronapyrrole C) [3]. The procedure of Shi et al. [19–21] was then used to effect an epoxidation of **8**. Application of substoichiometric amounts of oxidant (Oxone^®^) delivered the isomeric *mono*-epoxides **9** and **10** as the main constituents, along with minor amounts of the *bis*-epoxide **11**. Of note, on work-up **9** was observed to undergo partial cyclization to the desired tetrahydrofuran framework, a cyclization event that was driven to completion by treatment with camphorsulfonic acid under non-aqueous conditions. Subsequent heating in H_2_O/MeCN containing catalytic amounts of *p*-TsOH led to quantitative *N*-deprotection, while chromatographic purification of the resulting mixture provided heronapyrrole D (with an overall yield of 20% along with 8% of the previously known heronapyrrole C).

Cultivation and detection of heronapyrrole D: Saline broth inoculated with *Streptomyces* sp. (CMB-M0423) was cultivated and extracted as previously reported [1], after which it was analyzed by HPLC–DAD–MS–ESI(±) (Zorbax C_8_, gradient elution 90% to 10% H_2_O/MeCN with a constant 0.05% formic acid modifier). This analysis, supported by co-injection with authentic natural product standards, confirmed the presence of heronapyrroles A–C, and also detected an unidentified peak exhibiting the characteristic heronapyrrole (2-nitropyrrole) chromophore. HPLC–HRMS–ESI(+) analysis established a molecular formula (C_19_H_30_N_2_NaO_5_, Δmmu −0.2) for this unidentified heronapyrrole, consistent with that predicted for heronapyrrole D. Although semi-preparative HPLC fractionation returned a pure sample of this new heronapyrrole, the quantity available was insufficient for in depth structure elucidation. Fortunately, analytical HPLC–DAD comparisons between natural and synthetic samples of heronapyrrole D (see the Supporting Information File 1), together with optical rotation measurements [natural [α]_D_^22^ +7.5 (*c* 0.003, MeOH); synthetic [α]_D_^22^ +3.9 (*c* 0.1, MeOH)], established that heronapyrrole D was indeed a natural product, and that it possessed the structure and absolute configuration as proposed (see Supporting Information File 1, Figure S2). As with the known heronapyrroles A–C [1], heronapyrrole D exhibited growth inhibitory activity against the Gram-positive bacteria *Staphylococcus aureus* ATCC 25923 (IC_50_ 1.8 μM), *Staphylococcus epidermidis* ATCC 12228 (IC_50_ 0.9 μM) and *Bacillus subtilis* ATCC 6633 (IC_50_ 1.8 μM), but was inactive (IC_50_ > 30 μM) against the Gram-negative bacteria *Pseudomonas aeruginosa* ATCC 10145 and *Escherichia coli* ATCC 25922, and the fungus *Candida albicans* ATCC 90028.

## Conclusion

In conclusion, this study illustrates the importance of biosynthetic considerations to both natural products and synthetic chemistry. Biosynthetic hypotheses provide natural product chemists with a framework to challenge the plausibility of assigned structures, and inform and inspire synthetic chemists to develop novel biomimetic transformations and total syntheses. In this study we demonstrate a less well-appreciated benefit that arises from biosynthetic considerations, the ability to predict the occurrence of a new member of a rare class of natural product [22]. The initial discovery of the heronapyrroles A–C [1] prompted a successful biomimetic synthesis of heronapyrrole C [3], which in turn lead to speculation regarding the existence of heronapyrrole D. A biomimetic synthesis of heronapyrrole D was critical to establishing its status as a natural product, and to evaluating its antibacterial properties.

## Supporting Information

File 1 Experimental part and additional figures.
