# Supplementary material for: Heronapyrrole D: A case of co-inspiration of natural product biosynthesis, total synthesis and biodiscovery
Source: Beilstein J Org Chem. 2014 May 26;10:1228–32. doi: 10.3762/bjoc.10.121 (PMC4077399; doi:10.3762/bjoc.10.121)
Supplement: File 1 — Experimental part and additional figures. [file Beilstein_J_Org_Chem-10-1228-s001.pdf]

Supporting Information

for

Heronapyrrole D: A case of co-inspiration of natural product biosynthesis, total synthesis and biodiscovery

Jens Schmidt[1], Zeinab Khalil[2], Robert J. Capon*[2] and Christian B. W. Stark*[1]

Address: [1]Fachbereich Chemie, Institut für Organische Chemie, Universität Hamburg, Martin-Luther-King-Platz 6, 20146 Hamburg, Germany and [2]Institute for Molecular Bioscience, The University of Queensland, St. Lucia, QLD 4072, Australia

Email: Christian B. W. Stark - stark@chemie.uni-hamburg.de, Robert J. Capon - r.capon@uq.edu.au

* Corresponding authors

Experimental part and additional figures

Table of Contents

1 Experimental details performed by Capon group S3
1.1 General experimental details S3
1.2 Collection, cultivation, and characterisation of *Streptomyces* sp. (CMB-M0423) .. S3
1.3 Analytical cultivation and chemical profiling of *Streptomyces* sp. (CMB-M0423) S3
1.4 Preparative cultivation for *Streptomyces* sp. (CMB-M0423) S4
1.5 Chemical analysis of synthetic and natural heronapyrrole D (**6**) S4
1.6 Antibacterial assay S4
1.7 Antifungal assay S5

2 Experimental part performed by Stark group S5
2.1 General experimental details S5
2.2 Synthesis of (*S*,*E*)-1-((2*R*,5*R*)-5-(2-hydroxypropan-2-yl)-2-methyltetrahydrofuran-2-yl)-4-methyl-6-(5-nitro-1*H*-pyrrol-3-yl)hex-4-en-1-ol **6** S6
2.2.1 Compound characterization S7

List of Figures

Figure S1. HPLC–DAD–MS chromatogram of the crude extract from *Streptomyces* sp. (CMB-M0423) showing the secondary metabolites production including minor metabolites. Analytical gradient H_2O/MeCN containing 0.05% HCO_2H using a Zorbax C_8 column; absorbance at 360 nm. S7
Figure S2. HPLC–DAD (360 nm) chromatogram of samples of heronapyrrole D, (a) natural, (b) synthetic and (c) co-injection of natural and synthetic S8
Figure S3. Antibacterial activity of **6** S9
Figure S4. Antibacterial activity of **6** S10
Figure S5. ^{1}H NMR (500 MHz, CD_3OD) spectrum of **6** S11
Figure S6. ^{13}C (125 MHz, CD_3OD) spectrum of **6** S12

1 Experimental details performed by Capon group

1.1 General experimental details

Chiroptical measurements ($[\alpha]_D$) were obtained on a JASCO P-1010 polarimeter in a 100 × 2 mm cell. UV-visible spectra were obtained on a Cary 50 spectrophotometer in 1 cm quartz cells. NMR spectra were obtained on a Bruker Avance DRX600 spectrometer, in the solvents indicated and referenced to residual ^{1}H and ^{13}C signals in deuterated solvents. Electrospray ionization mass spectra (ESI–MS) were acquired using an Agilent 1100 Series separations module equipped with an Agilent 1100 Series LC/MSD in both positive and negative ion modes. High-resolution ESI–MS measurements were obtained on a Bruker micrOTOF mass spectrometer by direct infusion in MeCN at 3 μL/min using sodium formate clusters as an internal calibrant. HPLC was performed using an Agilent 1100 Series separation module equipped with an Agilent 1100 Series diode array and/or multiple wavelength detectors and an Agilent 1100 Series fraction collector, controlled using ChemStation Rev.9.03A and Purify version A.1.2 software.

1.2 Collection, cultivation, and characterisation of *Streptomyces* sp. (CMB-M0423)

Strain CMB-M0423 was isolated from a sediment sample (1 m depth) collected from Heron Island, Queensland. Wet sediment (1 mL) was transferred to a Falcon tube (5 mL) containing Ocean Nature seawater (4 mL of 33 g/L), shaken vigorously and heat-shocked at 55 °C for 8 min. An aliquot of the supernatant (50 μL) was dispersed across a solid phase agar isolation plate (M1 media) in the presence of 3.3% artificial ocean sea salt and incubated at 27 °C for 3 weeks. The plate was monitored on a regular basis for microbial growth. A pure culture was obtained for strain CMB-M0423 by repeated, single colony transfer on solid media.

1.3 Analytical cultivation and chemical profiling of *Streptomyces* sp. (CMB-M0423)

A single colony of CMB-M0423 was sub-sampled into seawater medium (100 mL of Ocean Nature seawater, 1% starch, 0.4% yeast extract and 0.2% peptone) and incubated at 27 °C for 15 d at 190 rpm. The culture was extracted with EtOAc (100 mL), and the organic phase concentrated in vacuo to yield an extract (2 mg) that was subsequently analyzed by HPLC–DAD–MS with conditions set as follows (Zorbax C_8 column, 150 × 4.6 mm, 5 μm, 1 mL/min, gradient from 90–10% H_2O/MeCN,

with an isocratic 0.05% formic acid modifier, over 15 min, with a hold at 100% MeCN for 5 min). Several peaks were detected and one of them (t_R = 9.1 min) exhibited the following *m/z* $[M - H]^-$ 365 (**6**). HRMS (ESI-TOF) *m/z* $[M + Na]^+$ calcd for $C_{19}H_{30}N_2NaO_5$ 389.2047; found: 389.2067.

1.4 Preparative cultivation for *Streptomyces* sp. (CMB-M0423)

Three Erlenmeyer flasks (2 L) containing M1 broth (400 mL; 1% starch, 0.4% yeast extract and 0.2% peptone) in the presence of 3.3% artificial ocean sea salt were inoculated with a starter culture (3 mL) of *Streptomyces* sp. (CMB-M0423) and incubated at 27 °C on a rotary shaker at 190 rpm for 15 days. The flasks were then extracted EtOAc (2 × 250 mL per flask) and the organic phases were combined and concentrated in vacuo to yield a combined EtOAc extract (60 mg). The EtOAc extract was sequentially triturated (8 mL aliquots) to recover hexane (15 mg), CH_2Cl_2 (40.2 mg) and MeOH (3.2 mg) soluble materials. The CH_2Cl_2 partition was further fractionated by HPLC (Zorbax C_8 column, 250 × 9.4 mm, 5 μm, 3 mL/min, gradient elution from 90–10% H_2O/MeOH over 40 min, with a hold at 100% MeOH for 5 min) to afford heronapyrrole D (**6**) (t_R= 15 min, 0.5 mg, 0.8%) [Note: % yields are determined on a mass to mass basis against the weight of EtOAc crude extract] (Figure S1).

1.5 Chemical analysis of synthetic and natural heronapyrrole D (6)

Different solutions of synthetic and natural heronapyrrole D (**6**) 0.05 mg/mL were dissolved in MeOH (1 mL) and the solutions were analyzed by HPLC-MS (Zorbax SB-C_8 5 μm 150 × 4.6 mm column, 1.0 mL/min, gradient elution from 90% H_2O/MeCN to 100% MeCN over 15 min followed by a 5 min hold at 100% MeCN, with isocratic 0.05% formic acid modifier) (Figure S2)

1.6 Antibacterial assay

The bacterium to be tested was streaked onto a tryptic soy agar plate and was incubated at 37 °C for 24 h. One colony was then transferred to fresh tryptic soy broth (15 mL) and the cell density was adjusted to 10^4–10^5 cfu/mL. The compounds to be tested were dissolved in DMSO and diluted with H_2O to give 300 μM stock solutions (10% DMSO). The stock solutions were then serially diluted with 10% DMSO to give final concentrations of 30 μM to 0.01 μM in 1% DMSO. An aliquot (20

µL) of each dilution was transferred to a 96-well microtiter plate and freshly prepared microbial broth (180 µL) was added to each well. The plates were incubated at 37 °C for 24 h and the optical density of each well was measured spectrophotometrically at 600 nm using POLARstar Omega plate (BMG LABTECH, Offenburg, Germany). Each test compound was screened against the Gram-negative bacteria *Escherichia coli* (ATCC 11775) and *Pseudomonas aeruginosa* (ATCC 10145) and the Gram-positive bacteria *Staphylococcus aureus* (ATCC 9144 and ATCC 25923) and *Bacillus subtilis* (ATCC 6633 and ATCC 6051). The IC_{50} value was calculated as the concentration of the compound or anticancer drug required for 50% inhibition of the cancer cells using Prism 5.0 from GraphPad Software Inc. (La Jolla, CA) (Figure S3 and Figure S4).

1.7 Antifungal assay

The fungus to be tested was streaked onto a Sabouraud agar plate and was incubated at 26.5 °C for 48 h. One colony was then transferred to fresh Sabouraud broth (15 mL) and the cell density was adjusted to 10^4–10^5 cfu/mL. Test compounds were dissolved in DMSO and diluted with H_2O to give a 300 µM stock solution (10% DMSO). The stock solution was then serially diluted with 10% DMSO to give final concentrations of 30 µM to 0.01 µM in 1% DMSO. An aliquot (20 µL) of each dilution was transferred to a 96-well microtiter plate and freshly prepared microbial broth (180 µL) was added to each well. The plates were incubated at 26.5 °C for 48 h and the optical density of each well was measured spectrophotometrically at 600 nm using POLARstar Omega plate (BMG LABTECH, Offenburg, Germany). Each test compound was screened against the fungus *Candida albicans* (ATCC 90028). The IC_{50} value was calculated as the concentration of the compound or anticancer drug required for 50% inhibition of the cancer cells using Prism 5.0 from GraphPad Software Inc. (La Jolla, CA).

2 Experimental part performed by Stark group

2.1 General experimental details

All reagents were used as purchased from commercial suppliers. Dry solvents were obtained from a MBraun solvent purification system (MB-SPS-800). All reactions were performed under an atmosphere of dry nitrogen. Reactions were monitored by thin-layer chromatography using silica pre-coated aluminium plates and stained with vanillin [vanillin (1 g), conc. H_2SO_4 (10 mL), AcOH (20 mL) ethanol (170 mL)] or ceric ammonium molybdate [phosphomolybdic acid (25 g),

$Ce(SO_4)_2\cdot 2H_2O$ (10 g), conc. H_2SO_4 (60 mL), H_2O (940 mL)]. Chromatographic purification was performed as flash chromatography on silica gel (particle size 0.040–0.063 mm). Yields refer to chromatographically purified and spectroscopically pure compounds. NMR spectra were obtained on a Bruker DRX-500 spectrometer (operating at 500 MHz for ^1H and 125 MHz for ^{13}C acquisitions) in the indicated solvents. Chemical shifts δ are reported in ppm with tetramethylsilane (TMS) or according to solvent resonance as the internal standard. Coupling constants *J* are given in Hertz (Hz). 2D NMR (H-COSY, HSQC, HMBC) data were used for the assignment of all final compounds. High-resolution ESI–MS measurements were obtained on an Agilent 6224 ESI–TOF mass spctrometer. IR spectra were recorded on a Bruker ALPHA FT-IR Platinum ATRspectrometer by attenuated total reflection. Absorbance frequencies $\tilde{\nu}$ are reported in reciprocal centimeters (cm^{-1}). Chiroptical measurements ($[\alpha]_D$) were obtained on a Krüss Optronic P8000 polarimeter at 589 nm using a 100 mm path-length cell in the solvent and concentration indicated. All compounds were named according to IUPAC rules. For simplicity, the numbering of the carbon atoms of a given structure did not follow the IUPAC rules.

2.2 Synthesis of (*S*,*E*)-1-((2*R*,5*R*)-5-(2-hydroxypropan-2-yl)-2-methyltetrahydrofuran-2-yl)-4-methyl-6-(5-nitro-1*H*-pyrrol-3-yl)hex-4-en-1-ol (6)

To a stirred solution of (*R*)-diol **8** (284 mg, 0.63 mmol, 1.00 equiv) in MeCN/$(CH_3O)_2CH_2$ (12 mL, 1:2) were added $Na_2B_4O_7\cdot 10H_2O$ in 4×10^{-4} M Na_2EDTA (0.05 M, 8.0 mL), *n*-Bu_4NHSO_4 (12 mg, 0.035 mmol, 0.055 equiv) and (+)-Shi ketone [S1] (97.0 mg, 0.38 mmol, 0.60 equiv). The mixture was cooled to 0 °C and solutions of Oxone® (658 mg, 1.07 mmol, 1.70 equiv) in Na_2EDTA (4×10^{-4} M, 5.0 mL) and of K_2CO_3 (609 mg, 4.41 mmol, 7.00 equiv) in H_2O (5 mL) were added separately via syringe pump over 90 min at 0 °C. The reaction mixture was diluted with H_2O (20 mL) and extracted with ethyl acetate (3 × 10 mL). The combined organic layers were dried over Na_2SO_4 and the solvent was removed under reduced pressure. The crude mixture was dissolved in dry toluene (20 mL) and (+)-camphorsulfonic acid (8.0 mg, 0.034 mmol, 0.05 equiv) was added at 0 °C. The solution was stirred for 1 h. Triethylamine (50 µL) was added and all volatiles were removed under reduced pressure (1×10^{-2} mbar). The residue was dissolved in MeCN (10 mL) and water (2 mL) was added. The solution was acidified with catalytic amounts of *p*-TsOH and heated to 50 °C for 1 h. Triethylamine (50 µL) was added after complete conversion and all volatile materials were removed under reduced pressure. Flash chromatography (66% ethyl acetate in

hexanes, silica) of the residue gave the title compound (46 mg, 20%) and heronapyrrole C (19 mg, 8%) as yellowish oils.

2.2.1 Compound characterization

(*S*,*E*)-1-((2*R*,5*R*)-5-(2-hydroxypropan-2-yl)-2-methyltetrahydrofuran-2-yl)-4-methyl-6-(5-nitro-1*H*-pyrrol-3-yl)hex-4-en-1-ol (**6**):

yellow oil; $[\boldsymbol{\alpha}]_{\mathbf{D}}^{\mathbf{20}}$ +3.9 (*c* 0.1, MeOH); **IR** (ATR) $\tilde{\nu}$ = 3362, 2972, 2927, 2872, 1503, 1452, 1356, 1294, 1266, 1119, 963, 886, 743 cm^{-1}; NMR (500 MHz, CD_3OD) δ_H 6.88 (d, *J* = 1.5 Hz, 1 H, H-5), 6.82 (d, *J* = 1.5 Hz, 1 H, H-3), 5.38 (t, *J* = 7.0 Hz, 1 H, H-7), 3.71 (dd, *J* = 9.5 Hz, *J* = 6.1 Hz, 1 H, H-15), 3.40 (dd, *J* = 10.3 Hz, *J* = 1.4 Hz, 1 H, H-11), 3.19 (d, *J* = 7.0 Hz, 2 H, H-6), 2.31-2.26 (m, 1 H, H-9a), 2.15–2.09 (m, 1 H, H-9b), 2.06–1.98 (m, 1 H, H-13a), 1.86–1.75 (m, 3 H, H-10a, H-14), 1.70 (s, 3 H, H-19), 1.64-1.59 (m, 1 H, H-13b), 1.42-1.34 (m, 1 H, H-10b), 1.15 (s, 3 H, H-17), 1.12 (s, 6 H, H-18, H-20) ppm; $^{\mathbf{13}}$**C** (125 MHz, CD_3OD) δ_c 138.7 (C-2), 137.4 (C-8), 127.6 (C-4), 123.9 (C-7), 123.4 (C-3), 111.3 (C-5), 88.0 (C-15), 86.7 (C-12), 76.9 (C-11), 72.2 (C-16), 37.3 (C-9), 35.1 (C-13), 30.8 (C-10), 27.7 (C-14), 26.4 (C-17), 26.1 (C-6), 25.1 (C-18/20), 22.7 (C-18/20), 16.1 (C-19) ppm; **HRMS** (ESI-TOF) *m/z* $[M+Na]^+$ calcd for $C_{19}H_{30}N_2NaO_5$ 389.2047; found: 389.2067.

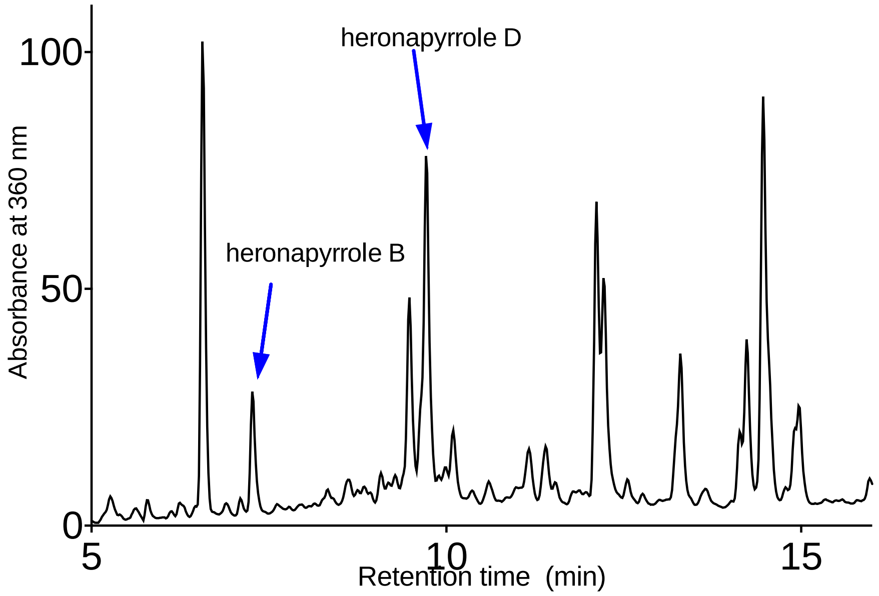

Figure S1: HPLC–DAD–MS chromatogram of the crude extract from *Streptomyces* sp. (CMB-M0423) showing the secondary metabolites production including minor metabolites. Analytical gradient H_2O/MeCN containing 0.05% HCO_2H using a Zorbax C_8 column; absorbance at 360 nm.

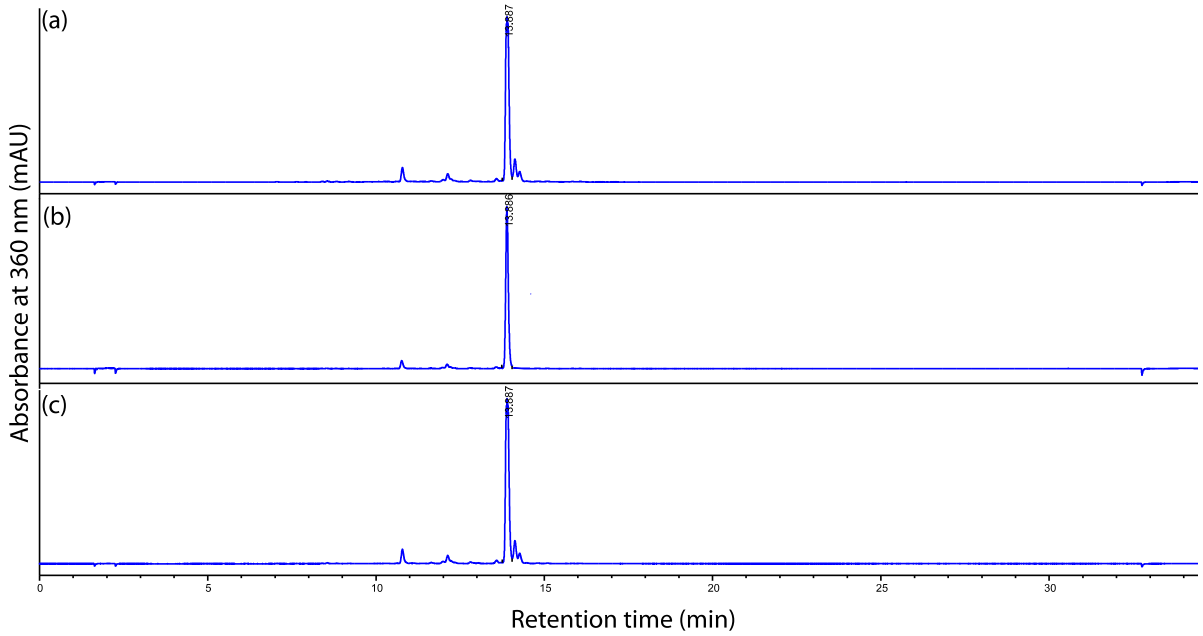

Figure S2: HPLC–DAD (360 nm) chromatogram of samples of heronapyrrole D, (a) natural, (b) synthetic and (c) co-injection of natural and synthetic.

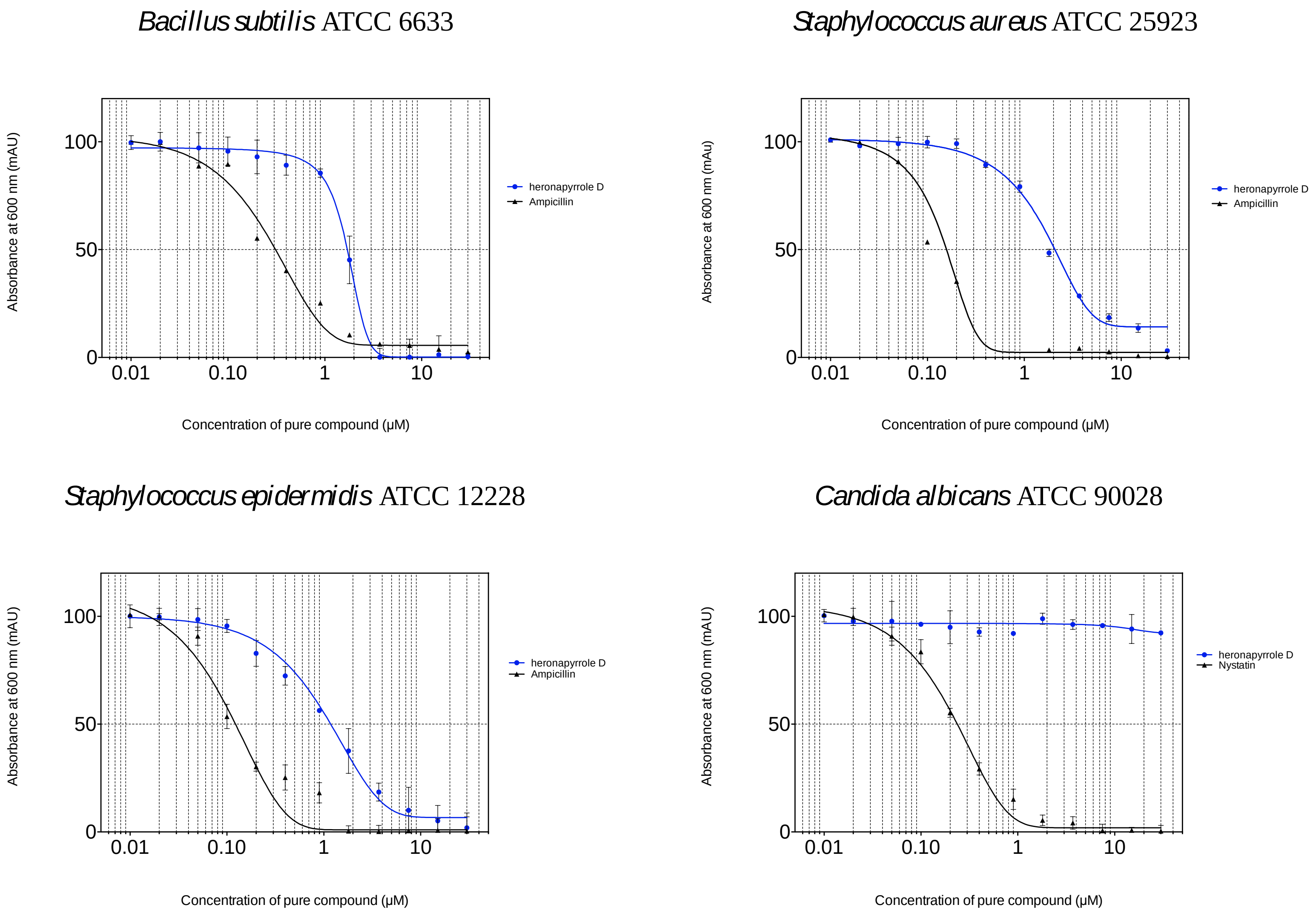

Figure S3: Antibacterial activity of **6** (average of two runs).

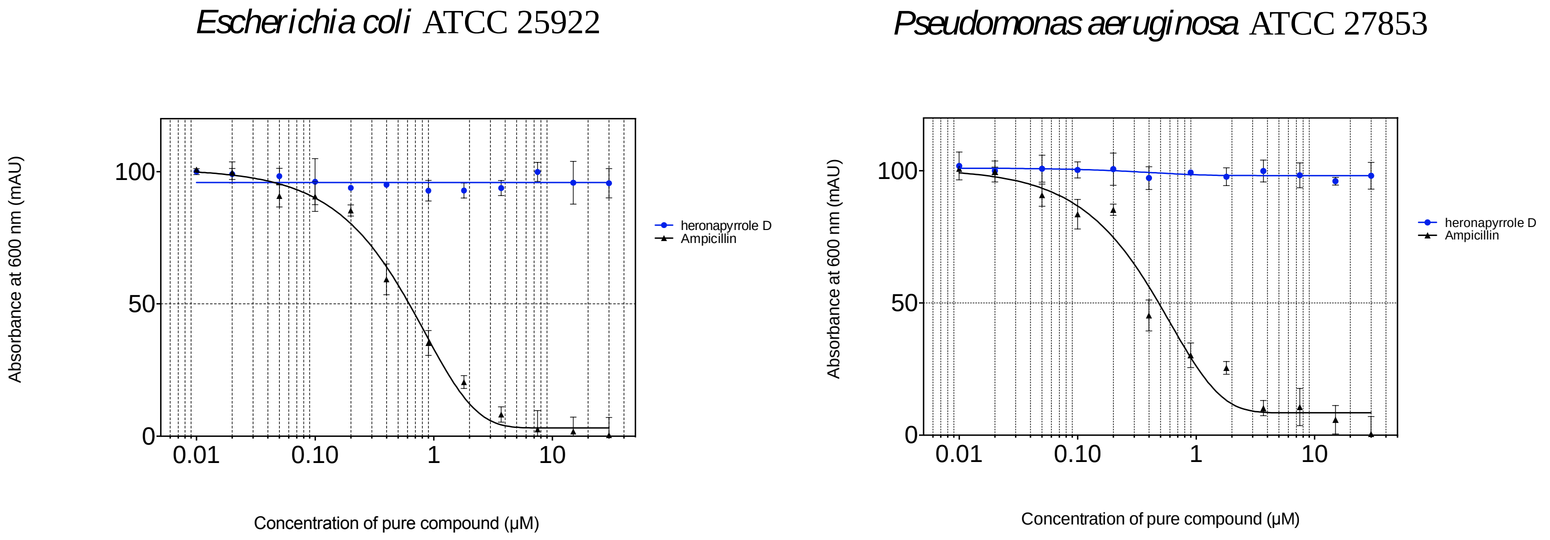

Figure S4: Antibacterial activity of **6** (average of two runs).

	***Escherichia coli* ATCC 25922**		***Pseudomonas aeruginosa* ATCC 27853**		***Staphylococcus aureus* ATCC 25923**		***Staphylococcus epidermidis* ATCC 12228**		***Bacillus subtilis* ATCC 6633**		***Candida albicans* ATCC 90028**	
**Compounds**	**MIC (µM)**	**IC_{50} (µM)**	**MIC (µM)**	**IC_{50} (µM)**	**MIC (µM)**	**IC_{50} (µM)**	**MIC (µM)**	**IC_{50} (µM)**	**MIC (µM)**	**IC_{50} (µM)**	**MIC (µM)**	**IC_{50} (µM)**
**Heronapyrrole D**	--	--	--	--	3.7	1.8	1.8	0.9	3.7	1.8	--	--
**Ampicillin**	0.9	0.6	0.5	0.36	0.2	0.15	0.2	0.1	0.4	0.25	--	--
**Nystatin**	--	--	--	--	--	--	--	--	--	--	0.4	0.2

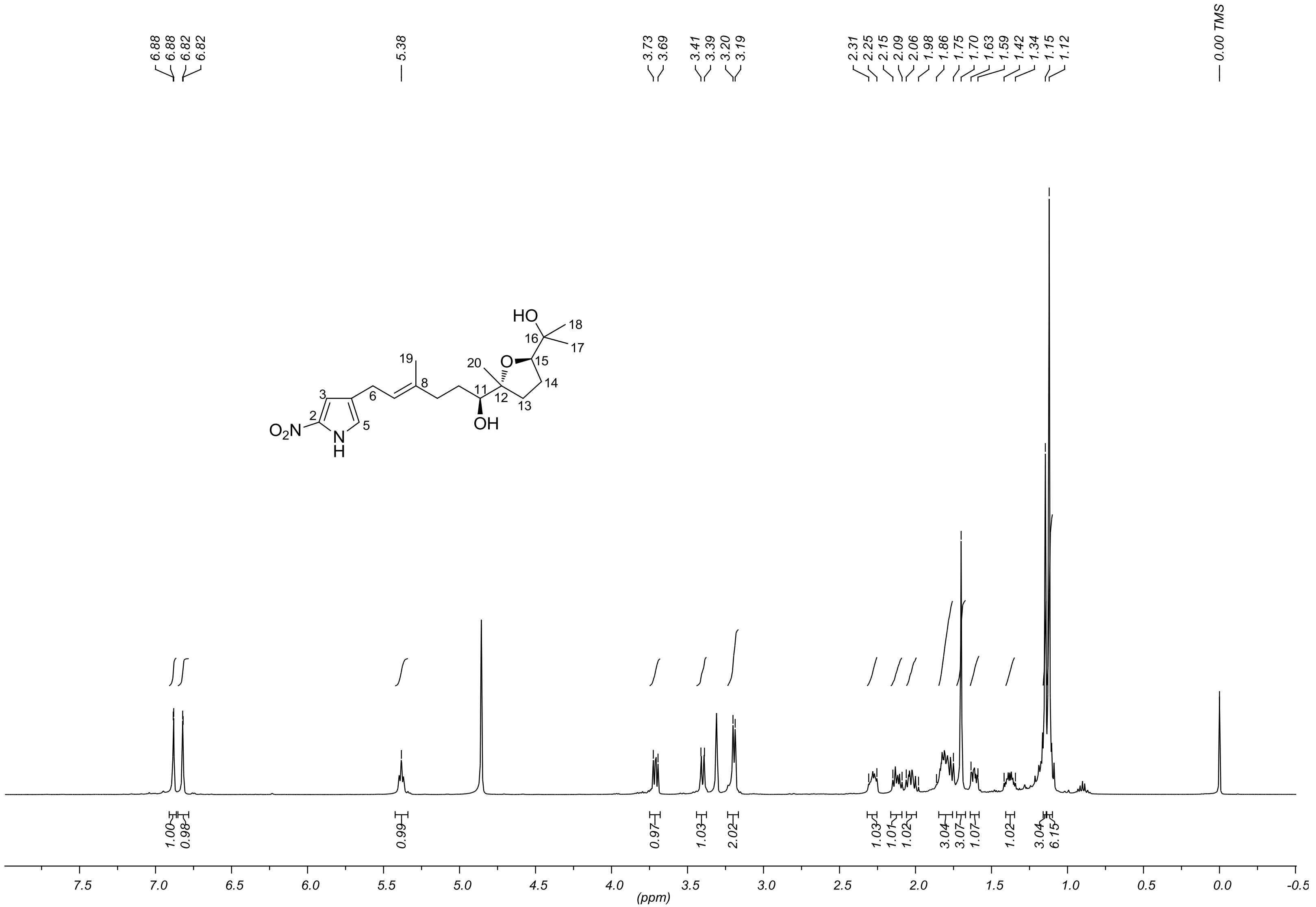

Figure S5: ^{1}H NMR (500 MHz, CD_3OD) spectrum of **6**.

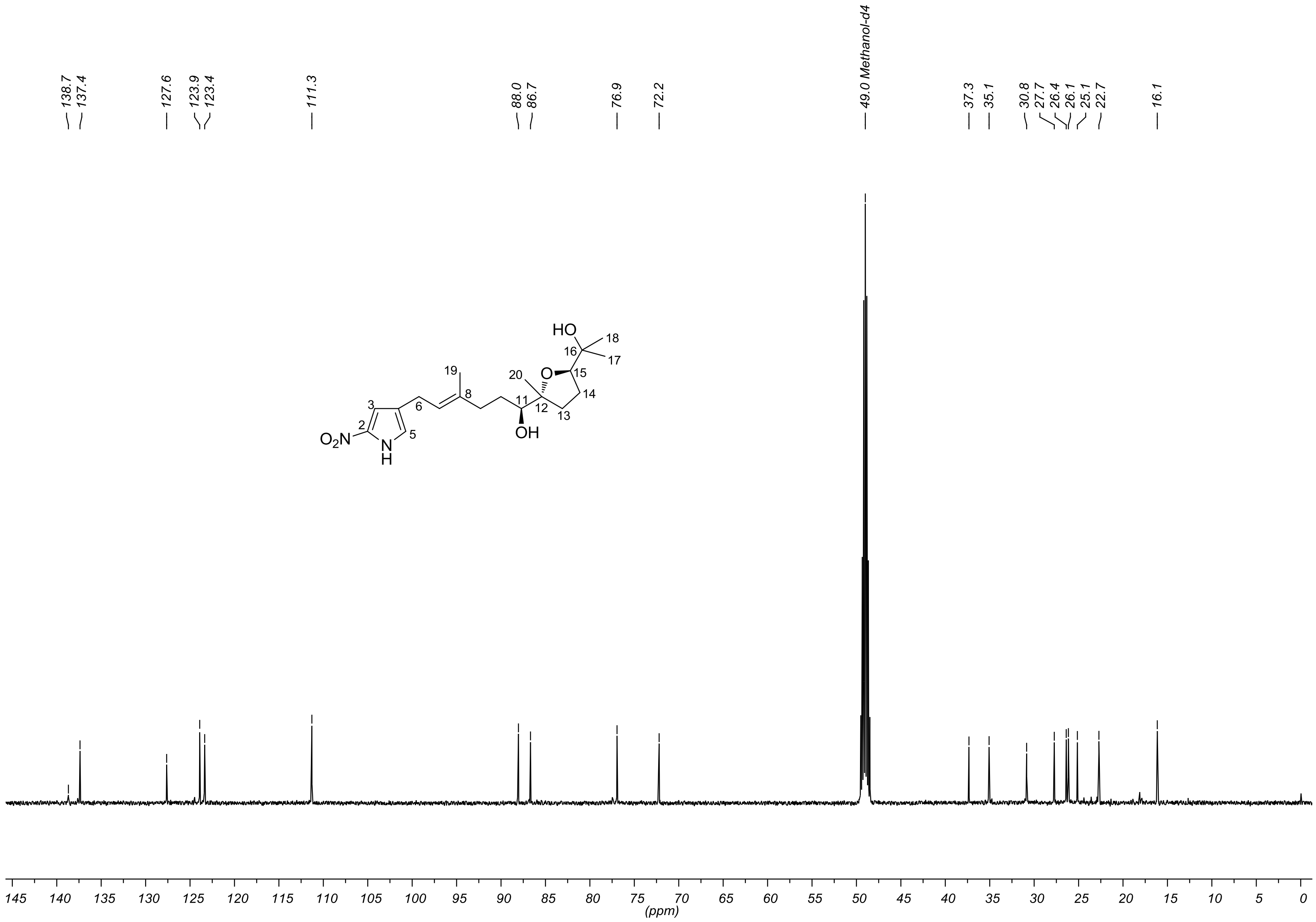

Figure S6: ^{13}C (125 MHz, CD_3OD) spectrum of **6**.

References

S1 (a) Zhao, M.-X.; Shi, Y. *J. Org. Chem.* **2006**, *71*, 5377–5379. (b) Wang, Z.-X.; Tu, Y.; Frohn, M.; Zhang, J.-R.; Shi, Y. *J. Am. Chem. Soc.* **1997**, *119*, 11224–11235. For a review, see: Frohn, M.; Shi, Y *Synthesis* **2000**, 1979–2000.
